# Simultaneous Detection of SARS-CoV-2 Nucleoprotein and Receptor Binding Domain by a Multi-Area Reflectance Spectroscopy Sensor

**DOI:** 10.3390/bios13090865

**Published:** 2023-09-01

**Authors:** Dimitra Tsounidi, Michailia Angelopoulou, Panagiota Petrou, Ioannis Raptis, Sotirios Kakabakos

**Affiliations:** 1 Immunoassays-Immunosensors Lab, Institute of Nuclear and Radiological Sciences and Technology, Energy and Safety, NCSR “Demokritos”, 15341 Aghia Paraskevi, Greece; dtsounidi@rrp.demokritos.gr (D.T.); mikangel@ipta.demokritos.gr (M.A.); ypetrou@rrp.demokritos.gr (P.P.); 2 Institute of Nanoscience and Nanotechnology, NCSR “Demokritos”, 15341 Aghia Paraskevi, Greece; i.raptis@inn.demokritos.gr

**Keywords:** reflectance spectroscopy, multi-analyte, SARS-CoV-2 proteins

## Abstract

The COVID-19 pandemic has emphasized the urgent need for point-of-care methods suitable for the rapid and reliable diagnosis of viral infections. To address this demand, we report the rapid, label-free simultaneous determination of two SARS-CoV-2 proteins, namely, the nucleoprotein and the receptor binding domain peptide of S1 protein, by implementing a bioanalytical device based on Multi Area Reflectance Spectroscopy. Simultaneous detection of these two proteins is achieved by using silicon chips with adjacent areas of different silicon dioxide thickness on top, each of which is modified with an antibody specific to either the nucleoprotein or the receptor binding domain of SARS-CoV-2. Both areas were illuminated by a single probe that also collected the reflected light, directing it to a spectrometer. The online conversion of the combined reflection spectra from the two silicon dioxide areas into the respective adlayer thickness enabled real-time monitoring of immunoreactions taking place on the two areas. Several antibodies have been tested to define the pair, providing the higher specific signal following a non-competitive immunoassay format. Biotinylated secondary antibodies and streptavidin were used to enhance the specific signal. Both proteins were detected in less than 12 min, with detection limits of 1.0 ng/mL. The assays demonstrated high repeatability with intra- and inter-assay coefficients of variation lower than 10%. Moreover, the recovery of both proteins from spiked samples prepared in extraction buffer from a commercial self-test kit for SARS-CoV-2 collection from nasopharyngeal swabs ranged from 90.0 to 110%. The short assay duration in combination with the excellent analytical performance and the compact instrument size render the proposed device and assay suitable for point-of-care applications.

## 1. Introduction

The coronavirus disease 2019 (COVID-19), caused by the severe acute respiratory syndrome coronavirus 2 (SARS-CoV-2) [1], has spread worldwide since 2019 and has affected more than 7.5 hundred million humans, as reported by the World Health Organization (WHO) [2]. Consequently, COVID-19 has caused a significant crisis in healthcare systems and has had a profound impact on society and the global economy [3]. This highlights the urgent need for timely and reliable diagnoses of viral infections, which is crucial for effective treatment and the efficient management of public health resources during a pandemic [4,5]. Several methods have been employed for SARS-CoV-2 detection, with quantitative Reverse Transcription PCR (qRT-PCR) being the gold standard [6,7,8]. Although qRT-PCR tests are highly sensitive and specific and capable of identifying the virus at the early stages of infection, they can only be conducted in certified laboratories with expensive equipment and well-trained personnel. Moreover, despite qRT-PCR tests taking a few hours to complete, the turnaround time from sample collection to obtaining results exceeds 24 h or even several days, leading to a significant delay in COVID-19 diagnosis [6,9]. The same limitation applies to alternative molecular tests, such as Reverse Transcription Loop-mediated isothermal Amplification (RT-LAMP), which are more suitable for point-of-care application than the standard qRT-PCR [10,11]. These methods, however, require RNA extraction from the samples, which adds to the complexity and the time required for analysis. Immunochemical methods, including enzyme-linked immunosorbent assay (ELISA) and chemiluminescence immunoassays [12], have also been widely used for SARS-CoV-2 detection. However, these methods have limitations regarding the duration and cost of the assays as well as the requirement for special equipment or sample pretreatment [13].

Point-of-care tests have been proposed as the best alternative solution for COVID-19 diagnosis. These tests should be rapid and cost-effective as well as suitable for performance outside clinical laboratories, with short turnaround times and minimal user intervention. Additionally, they should be characterized by high sensitivity and accuracy [13,14]. In this regard, several analytical methods based on lateral flow immunoassays (LFIA) have been developed for the detection of SARS-CoV-2 [15,16]. These methods are known for their speed (results obtained in 10–30 min) and can be performed by non-trained personnel [15,16]. However, LFIAs provide qualitative or semi-quantitative results and exhibit lower sensitivity and specificity compared to standard immunochemical methods [17]. On the other hand, biosensors could provide a reliable solution for point-of-care SARS-CoV-2 detection. They offer a combination of rapid, sensitive, and selective detection, along with quantitative results, low consumable costs, and instrument portability [18,19,20,21,22]. The biosensing methods so far exploited for COVID-19 diagnosis rely on electrochemical [23] or optical transducers [24,25]. The Receptor-Binding Domain (RBD) of the spike protein (S) and the nucleocapsid protein (NP) are considered the most appropriate antigens among the structural viral proteins for detecting SARS-CoV-2 in clinical samples [21,22]. RBD is part of the S1 subunit of the spike protein, a glycoprotein present on the virus surface. Its main role is to facilitate the virus entry into host cells through its binding to the angiotensin-converting enzyme 2 (ACE2) on the surface of human cells [26,27,28]. On the other hand, NP is responsible for packaging the viral genome RNA into ribonucleocapsid and plays a role in the virus transcription and replication cycle, as well as in the host cellular response to viral infection [29,30]. In addition, NP is more abundant than S protein and less prone to mutations [22], and it can be detected in nasopharyngeal swabs one day before the appearance of clinical symptoms [31]. Hence, different biosensing approaches have been proposed for the determination of RBD and NP, implementing electrochemical [32,33] or optical [34,35,36,37] transducers based mainly on the immunochemical detection of these two SARS-CoV-2 proteins. To the best of our knowledge, however, none of the proposed biosensors enables the simultaneous detection of both RBD and NP proteins, which could contribute to a more accurate and reliable diagnosis of COVID-19, minimizing false-negative results.

Thus, the aim of this work was to apply an optical label-free immunosensor for the rapid and simultaneous determination of RBD and NP of SARS-CoV-2 in extracts from nasopharyngeal swabs without any pretreatment. The developed immunosensor is based on Multi Area Reflectance Spectroscopy (MARS), a detection approach that belongs to the family of reflectometric transduction principles. These optical detection methods have evolved from ellipsometry and have acquired multiple configurations and applications for the label-free monitoring of biomolecular interactions [38,39]. The MARS detection approach has previously been applied for the simultaneous detection of other analytes [40,41]. In MARS technology, silicon chips with two areas of silicon dioxide layer of different thicknesses are implemented. Each area is modified with a specific antibody against one of the targeted analytes. Simultaneous illumination of these two sensing areas results in a collected reflected interference spectrum that combines the spectra from both areas. The collected spectrum is processed in real-time by a dedicated software, allowing the monitoring of reactions taking place in the two areas as an increase in the respective biomolecular adlayer thickness. For the simultaneous determination of RBD and NP, a two-step sandwich immunoassay was developed. This includes reaction of the specific capture antibodies immobilized on the two sensing areas, with the RBD or NP in the calibrators or the samples, and the reaction of the immunoadsorbed analyte molecules with a mixture of specific detection antibodies. Several antibodies were tested in order to select a pair of capture and detection antibodies for each protein that provided the highest specific signal in combination with the lowest non-specific signal. To enhance the signal and reduce assay duration, biotinylated secondary antibodies specific to the detection antibodies, in combination with streptavidin, were employed. The dual-analyte assay development was performed under the restriction that common conditions had to be adopted for both analytes, aiming at the highest possible detection sensitivity and the shortest assay duration. Moreover, to create a practical solution for point-of-care diagnostics, the developed immunosensor was incorporated into a compact bench-top device. This device encompasses all the necessary electronic and optical components required to execute the assay. Its design allows for reliable automated measurements at the point-of-care, providing convenience and accuracy. To evaluate the performance of the dual-analyte immunosensor, spiked samples prepared in extraction buffer from a commercial self-test kit for SARS-CoV-2 collection from nasopharyngeal swabs were used.

## 2. Materials and Methods

### 2.1. Materials

Recombinant SARS-CoV-2 S1 subunit protein RBD domain and nucleocapsid protein, derived from *E. coli*, were obtained from RayBiotech Life, Inc. (Peachtree Corners, GA, USA). All antibodies used against SARS-CoV-2 spike protein RBD and nucleocapsid protein were provided from HyTest Ltd. (Turku, Finland). These antibodies included three recombinant full-sized chimeric monoclonal antibodies expressed in mammalian cells, with wild type variable domains of rat monoclonal antibody and human IgG1 constant domains, against the SARS-CoV-2 spike protein RBD domain (codes RBD5305, RBD5308, RBD5313); one recombinant rabbit full-sized monoclonal antibody expressed in mammalian cells against the SARS-CoV-2 spike nucleoprotein (code C706); three hybridoma clones derived from the hybridization of Sp2/0 myeloma cells with spleen cells of Balb/c mice immunized with recombinant SARS-CoV-2 Spike RBD domain (codes R107, RBD1106, RBD1125); and six hybridoma clones derived from Balb/c mice immunized with recombinant SARS-CoV-2 nucleoprotein (codes C518, NP1503, NP1510, NP1516, NP1517, NP1529). Anti-human IgG Fc-specific antibody produced in goat and streptavidin were obtained from Thermo Fisher Scientific (Waltham, MA, USA). Anti-rabbit IgG (whole molecule) antibody produced in goat and (3-aminopropyl)triethoxysilane (APTES) were obtained from Sigma-Aldrich (Darmstadt, Germany). Bovine serum albumin (BSA) was purchased from Acros Organics (Geel, Belgium). All other reagents were from Merck (Darmstadt, Germany). Biotinylation of goat anti-rabbit IgG and goat anti-human IgG Fc-specific antibody was performed following a protocol described in the literature [42]. Four-inch device-quality Si wafers were purchased from Si-Mat Germany (Kaufering, Germany). The buffer used for extraction of SARS-CoV-2 virus from nasopharyngeal swabs was obtained from the SARS-CoV-2 self-test kit (COVID-19 Antigen Rapid Test; Clongene, Hangzhou, China).

### 2.2. Instrumentation

The optical module of the measurement set-up consists of a visible/near-infrared light source, a miniaturized spectrometer, and a reflection probe that encompasses six illumination fibers with a core diameter of 600 μm arranged at the periphery of the probe. A seventh central fiber of the same diameter collects the reflected light and guides it to the spectrometer. The miniaturized spectrometer (Maya 2000 Pro, 16-bit A/D) is tuned to operate in the 450–650 nm spectral range. The Si chip designed for dual-analyte detection measures 5 mm × 15 mm featuring SiO_2_ areas with thicknesses of 775 and 990 nm, respectively. Each SiO_2_ area has dimensions 4 mm × 2 mm, and each chip contains two sets of areas with the same thickness (i.e., four 4 cm × 2 cm areas in total). For the continuous delivery of reagent solutions, the biofunctionalized chip is covered by a custom-designed microfluidic cell (Jobst Technologies GmbH, Freiburg, Germany). This cell is connected to both a rotating sampler and a programmable peristaltic micropump. The assay starts by placing the biofunctionalized chip in a docking station that provides automatic alignment and a stable position in relation to the reflection probe. The reader used is accompanied by software that controls the micropump, the reagents’ carousel, and the sampler, thus enabling the automated execution of the assay [40]. An image of the reader along with the PC used to run the software is provided in Figure S1. The software continuously collects and analyses the reflectance spectrum recorded from the two sensing areas (at a rate of 1 spectrum per second), providing the evolution of effective biomolecular adlayer thickness at each of the SiO_2_ areas throughout the assay. The thickness values obtained for the calibrators are used to construct preloaded calibration curves, based on which the software automatically provides the concentration of the analytes in the samples after the end of each measurement.

### 2.3. Chemical Activation and Biofunctionalization of the Chip

The MARS chips were initially cleaned by subjecting them to successive 10-min sonication baths of acetone and 2-propanol. After drying with nitrogen, they were further treated with Piranha solution (1:1 H_2_SO_4_/30% *v*/*v* H_2_O_2_) for 20 min. Following thorough washing with distilled water, the chips were immersed in a 2% *v*/*v* APTES solution in ethanol for 1 h. They were then gently washed with ethanol and dried with nitrogen. Finally, the chips were cured at 120 °C for 20 min and stored at room temperature (RT) in a desiccator until use.

For the biofunctionalization, the two SiO_2_ areas were spotted using the BioOdyssey Calligrapher Mini Arrayer (Bio-Rad Laboratories, Inc., Hercules, CA, USA) with a 200 μg/mL anti-RBD antibody solution (code R107) and a 200 μg/mL anti-NP antibody solution (code C518), respectively, both prepared in 0.05 M carbonate buffer, pH 9.2. Multiple overlapping spots with a diameter of 400 μm were deposited to cover the chip area illuminated by the reflection probe. After overnight incubation at RT in a humidity chamber (75% humidity), a blocking step was performed by immersing the biofunctionalized chips in the blocking solution (1% *w*/*v* BSA in 0.1 M NaHCO_3_, pH 8.5) for 2 h at RT. The chips were then washed with 0.01 M Tris-HCl, pH 8.5 (washing solution), dried with nitrogen, and stored at 4 °C until use.

### 2.4. Simultaneous Immunodetection of RBD and NP Biomarkers with MARS Biosensor

**Figure 1:**
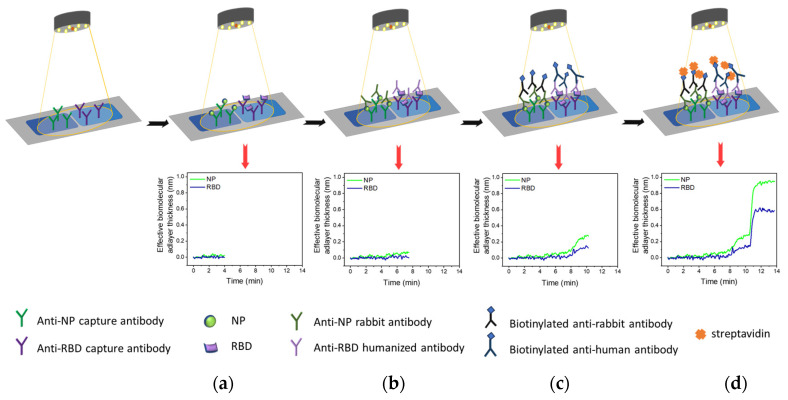
Schematic of the immunoassay procedure for the simultaneous determination of RBD and NP with the MARS biosensor (upper part) along with the expected sensor responses during the successive immunoassay steps (lower part). These steps included: (**a**) reaction of RBD and NP molecules with the immobilized on the chip capture antibodies, (**b**) reaction of detection antibodies with the immunoadsorbed analyte molecules, (**c**) reaction of the immunoadsorbed detection antibodies with the respective biotinylated secondary antibodies, and (**d**) reaction with streptavidin for signal enhancement.

Prior to immunoassay, each biofunctionalized chip was assembled with the microfluidic cell, placed in the docking station of the device, and connected with the reagents handling the module and the micropump. The biochip was then equilibrated with assay buffer (0.05 M Tris-HCl, pH 7.8, 0.9% *w*/*v* NaCl, 0.5% *w*/*v* BSA) for a few mins before running the RBD and NP calibrators prepared in assay buffer or the samples for 3 min. This was followed by a 3-min run of detection antibodies solution in assay buffer (2.5 μg/mL of anti-RBD antibody (code RBD5308) and 2.5 μg/mL of anti-NP antibody (code C706)). Subsequently, a solution containing 10 μg/mL of biotinylated anti-human IgG and 10 μg/mL of biotinylated anti-rabbit IgG antibodies in assay buffer was run over the biochip for 3 min. Finally, a 10 μg/mL streptavidin solution in assay buffer was run for 2 min. The assay was completed by running assay buffer for 1 min. All solutions run at a constant flow rate of 40 μL/min. The immunoassay procedure is schematically depicted in Figure 1.

## 3. Results

### 3.1. Assay Development and Optimization

**Figure 2:**
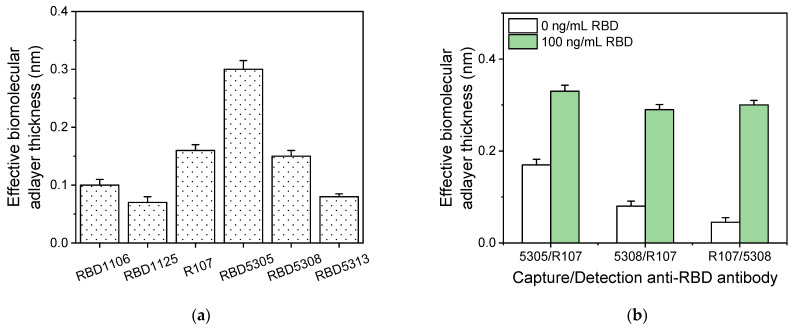
Selection of anti-RBD antibodies. (**a**) Responses obtained from chips coated with a 200 μg/mL RBD solution upon running different antibodies raised against RBD. The concentration of all antibodies was 1 μg/mL, and the solutions were run for 15 min at 40 μL/min. Each column is the mean value of three measurements ± SD. (**b**) Responses obtained for 0 (white columns) and 100 ng/mL RBD solutions (green columns) using the following antibody pairs as capture and detection, respectively: RBD5305-R107, RBD5308-R107, and R107-RBD5308. In all cases, a secondary antibody was used for the signal enhancement. Each column is the mean value of three measurements ± SD. The combination of anti-RBD R107 as the capture antibody and anti-RBD5308 as the detection antibody provided the highest analytical signal along with a low non-specific signal.

The simultaneous detection of SARS-CoV-2 virus RBD and NP proteins with the MARS biosensor was based on non-competitive immunoassays using pairs of antibodies specific for the two targeted analytes. To select antibodies with high binding affinity for the two analytes, several antibodies were initially tested in a direct binding assay using chips modified with the two targeted analytes. In this context, a solution of all of the candidate antibodies with a concentration of 1 μg/mL was run for 15 min over chips modified either with RBD or NP. The signals obtained for all antibodies specific for the RBD are presented in Figure 2a. As shown, the antibodies with codes RBD5305, RBD5308, and R107 provided the highest signal values. The RBD5305 and RBD5308 antibodies are humanized rat antibodies, whereas R107 is a mouse monoclonal antibody. Thus, the combinations tested were RBD5305-R107, RBD5308-R107, and R107-RBD5308 so as to be able to use, for signal enhancement, a secondary antibody (anti-mouse or anti-human antibody) that does recognize the capture antibody. The selection criterion for the antibody pairs was based on the signal obtained for the zero calibrator (non-specific binding signal) as well as the signal obtained for a calibrator containing a fixed concentration of the analyte (specific signal). As shown in Figure 2b, the combination of anti-RBDR107 as the capture antibody and anti-RBD5308 as the detection antibody provided the highest analytical signal along with a negligible non-specific signal, and was therefore selected.

**Figure 3:**
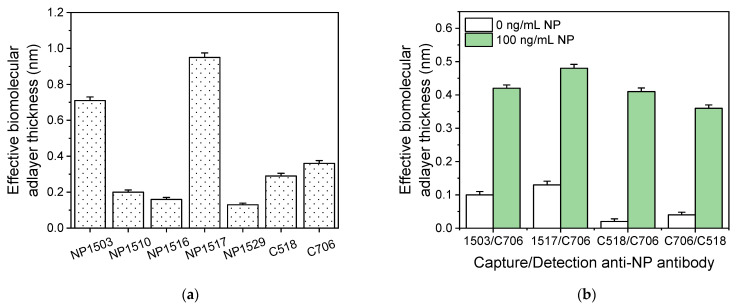
Selection of anti-NP antibodies. (**a**) Responses obtained from chips coated with a 200 μg/mL NP solution upon running different antibodies raised against NP. The concentration of all antibodies was 1 μg/mL, and the solutions were run for 15 min at 40 μL/min. Each column is the mean value of three measurements ± SD. (**b**) Responses obtained for 0 (white columns) and 100 ng/mL NP solutions (green columns) using the following antibody pairs as capture and detection, respectively: NP1503-C706, NP1517-C706, C518-C706, and C706-C518. In all cases, a secondary antibody was used for the signal enhancement. Each column is the mean value of three measurements ± SD. The combination of anti-NP C518 as the capture antibody and anti-NP C706 as the detection antibody provided the highest analytical signal along with a low non-specific signal.

Regarding the antibodies against NP, the results obtained for their direct binding to NP-modified chips are presented in Figure 3a. As shown, the antibodies with codes NP1503, NP1517, C518, and C706 provided high signals. Thus, NP1503, NP1517, and C518 are the mouse monoclonal antibodies that were tested in combination with the rabbit monoclonal antibody, C706, as detection antibody, and C706 was tested as capture antibody in combination with C518 as detection antibody in order to select the best pair. It was found that the highest specific-to-non-specific signal ratio was achieved using the anti-NP C518 antibody as the capture antibody and the anti-NP C706 as the detection antibody. An anti-rabbit IgG antibody was used for signal enhancement in this case (Figure 3b).

**Figure 4:**
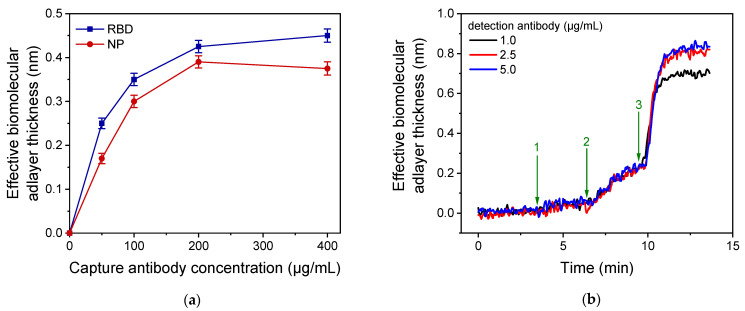
(**a**) Optimization of the concentration of capture antibodies. Signal values obtained for a 100 ng/mL RBD calibrator (blue squares) and a 100 ng/mL NP calibrator (red circles) from chips coated with capture antibodies against RBD or NP, respectively, at concentrations ranging from 50 to 400 μg/mL. Each point is the mean value of three measurements ± SD. A 200 μg/mL concentration of both anti-RBD and anti-NP antibodies was selected for coating of the MARS chips. (**b**) Optimization of anti-NP detection antibody concentration. Real-time responses obtained for a 20 ng/mL NP calibrator from chips coated with 200 μg/mL of anti-NP antibody C518 used for detection of the anti-NP antibody C706 at concentrations of 1.0 (black line), 2.5 (red line), and 5.0 μg/mL (blue line). The arrows indicate the solutions that run over the chip: start to arrow 1 assay buffer; arrow 1 to 2, NP calibrator; arrow 2 to 3, detection antibody; and arrow 3 to end, secondary antibody. Maximum plateau values were obtained for detection antibody concentration equal to or higher than 2.5 μg/mL.

Subsequently, the optimum concentration for the two capture antibodies was determined using chips coated with anti-RBD antibody R107 or anti-NP antibody C518 solutions, with concentrations ranging from 50 to 400 μg/mL. All chips were tested using a 100 ng/mL RBD or NP calibrator, respectively, and the responses obtained are presented in Figure 4a. Regarding the RBD assay, the sensor response increased as the antibody concentration in the coating solution increased, reaching maximum plateau values at concentrations equal to or higher than 200 μg/mL. Therefore, this concentration was selected for the capture antibody against RBD in the final immunoassay protocol. Similarly, the optimum concentration of the capture anti-NP antibody was determined using a 100 ng/mL NP calibrator, and it was found that a concentration of 200 μg/mL provided maximum signal values (Figure 4a). In this case, a slight signal decrease was observed when a higher anti-NP antibody concentration was used (400 μg/mL), which can be ascribed to the very close packaging of immobilized antibody molecules, leading to reduced antigen binding due to steric hindrance effects. Using the selected capture antibody concentrations for RBD and NP, the concentrations of the detection antibodies were also optimized. It was found that maximum plateau values were provided for detection antibody concentrations equal to or higher than 2.5 μg/mL for both the RBD and NP immunoassays (Figure 4b). Thus, this concentration was selected for the final protocol.

Using the optimal capture and detection antibody concentrations, the duration of each step of the RBD and NP immunoassays was optimized with the aim to achieve the shortest possible assay duration. Additionally, the secondary antibodies were replaced by biotinylated ones and an additional reaction step with streptavidin was introduced to further enhance the signal and thus increase the detection sensitivity.

**Figure 5:**
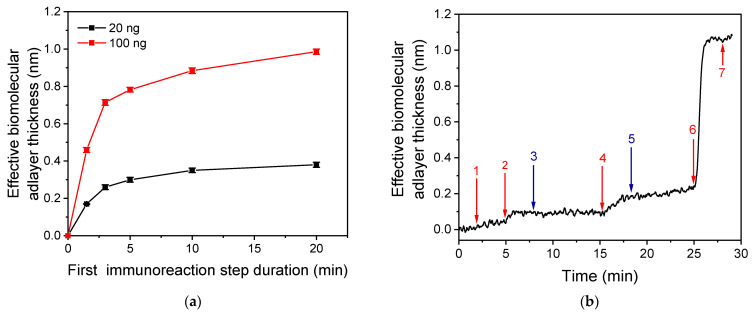
Optimization of assay steps duration. (**a**) Signal values obtained for a 20 (red squares) and a 100 ng/mL RBD calibrator (black squares) running from 1.5 to 20 min over a chip coated with an anti-RBD antibody followed by a 3-min reaction with a 2.5 μg/mL detection anti-RBD antibody solution, a 3-min reaction with a 10 μg/mL biotinylated anti-human IgG antibody solution, and a 2-min reaction with a 10 μg/mL streptavidin solution. Each point is the mean value of three measurements ± SD. A 3-min reaction of calibrators with the capture antibody provided adequate signal, equal to 70% of the signal obtained for a 20-min reaction. (**b**) Real-time response obtained for a 100 ng/mL RBD calibrator running for 3 min over a chip coated with an anti-RBD antibody (arrow 1 to 2) followed by a 10-min reaction with a 2.5 μg/mL detection anti-RBD antibody solution (arrow 2 to 4), a 10-min reaction with a 10 μg/mL biotinylated anti-human IgG antibody solution (arrow 4 to 6), and a 2-min reaction with a 10 μg/mL streptavidin solution (arrow 6 to 7). Arrows 3 and 5 correspond to the signal obtained after 3-min running of the detection antibody and of the biotinylated anti-human IgG antibody, respectively.

Figure 5a shows the signals obtained for a 20 ng/mL and a 100 ng/mL RBD calibrator for different durations of the first immunoassay step, which involves the reaction of analyte molecules with the capture antibody immobilized onto the chip. The presented responses correspond to the signal received after reaction of immunoadsorbed analyte molecules with the detection antibody for 3 min (second immunoassay step), and reaction of the immunoadsorbed detection antibody with the secondary antibody for 3 min (enhancement step). As shown, the signals obtained for both calibrators increased as the duration of the first immunoreaction step increased from 1.5 to 20 min. However, when the duration of the first immunoreaction step was increased from 3 to 20 min, the signal obtained for the 20 ng/mL and 100 ng/mL calibrators increased by approximately 40% and 45%, respectively. Nonetheless, the signals received for a 3-min first immunoreaction duration were adequate even for the calibrator with the lower concentration. Thus, considering the need for rapid measurements, a 3-min duration was selected for the first immunoassay step.

Regarding the reaction of the detection antibody with the immunoadsorbed RBD molecules, a 3-min duration was also adopted in the final protocol. As shown in the real-time signal response of Figure 5b, the maximum plateau signal values were received after 3 min of reaction (arrow 3), and no further signal increase was observed when this step duration was increased to 10 min (arrow 4). Concerning the duration of the biotinylated secondary antibody (anti-human IgG) reaction with the immunoadsorbed detection antibody, a 3-min duration was selected (Figure 5b, arrow 5). At this point, almost 70% of the maximum plateau signal achieved after 10 min of reaction (Figure 5b, arrow 6) was obtained. Finally, the reaction with the streptavidin was completed in less than 2 min, and this reaction time was adopted in the final protocol to take full advantage of signal enhancement effect of streptavidin. Similar results were obtained for the NP immunoassay, enabling their simultaneous determination. Regarding the signal enhancement achieved using the biotinylated secondary antibody in combination with streptavidin, as shown in Figure 5b, the signal received from the primary immunoreaction increased approximately 2.5 times after reaction with the biotinylated secondary antibody (from 0.092 to 0.24 nm). The reaction with streptavidin led to a further 4.4-fold increase of the signal from 0.24 to 1.06 nm, amounting to a 11.5-fold increase of the signal from the primary immunoreaction. The spectacular signal increase observed upon reaction with streptavidin, as compared to that achieved with the biotinylated antibody, can be ascribed to the fact that the affinity constant of streptavidin for binding with biotin is six orders of magnitude higher compared with the affinity constant of the antibody-antigen reaction. Furthermore, this result could be due to the multiple site labeling of secondary antibodies with biotin, which enable the binding of several streptavidin molecules per molecule of immunoadsorbed secondary antibody.

**Figure 6:**
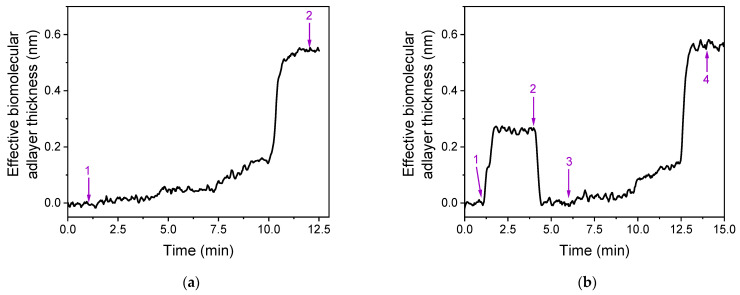
Effect of SARS-CoV-2 extraction buffer from nasopharyngeal swab samples on the sensor response. (**a**) Real-time response obtained for a 50 ng/mL RBD calibrator prepared in assay buffer. Arrows 1 and 2 indicate the time frame for which the sensor response for the particular calibrator is calculated. (**b**) Real-time response obtained for a 50 ng/mL RBD calibrator prepared in extraction buffer from a commercial kit for self-test of SARS-CoV-2. Arrows 1 to 2 indicate the introduction of calibrator solution, arrows 2 to 3 indicate the following washing step, arrows 3 to 4 the reaction with the detection antibody, the biotinylated secondary antibody, and streptavidin. The sensor response for the calibrator was calculated by subtracting from the value at arrow 4 the one corresponding to arrow 1. The signal obtained for the calibrator prepared in extraction buffer was identical to that obtained for the same calibrator prepared in assay buffer.

All optimization experiments were conducted with calibrators prepared in assay buffer. However, since the intended application of the dual analyte detection is for SARS-CoV-2 virus in extracts of nasopharyngeal swab samples, it was necessary to evaluate the sensor response to calibrators prepared in extraction buffer from a commercially available self-test kit for SARS-CoV-2 antigen detection (COVID-19 Antigen Rapid Test, Clongene, Hangzhou, China) and compare the signals obtained with those from calibrators prepared in assay buffer. Figure 6 illustrates the real-time signal responses for a 50 ng/mL RBD calibrator prepared in assay buffer (Figure 6a) and in the extraction buffer (Figure 6b). As shown in Figure 6b, the introduction of the extraction buffer (indicated by arrow 1) caused an abrupt change in the real-time signal, likely due to a difference in refractive index compared to the assay buffer. However, after a 2-min washing step with assay buffer (Figure 6b, arrows 2 to 3), the signal equilibrated, and the sensor response during the next immunoassay steps could be observed in real-time without interference from the matrix. The difference in signal after the end of the immunoassay (Figure 6b, arrow 4) compared to the signal prior to the immunoassay (Figure 6b, arrow 1) was identical to that obtained with the calibrator prepared in assay buffer (Figure 6a, arrows 1 to 2). This observation was consistent for other RBD calibrators as well as for the NP assay. Therefore, the developed dual-analyte immunosensor could be used for the determination of RBD and NP in nasopharyngeal swab extracts by introducing a short washing step after the first immunoreaction, without significantly increasing the overall assay duration. Additionally, calibration could be performed using analyte solutions prepared in assay buffer.

**Figure 7:**
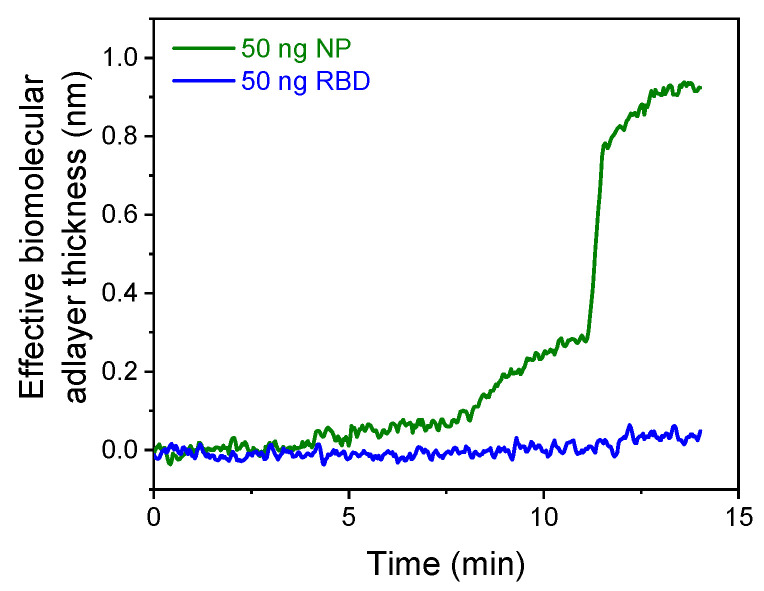
Real-time responses obtained from the area coated with the anti-NP mAb (green line) and the area coated with the anti-RBD mAb (blue line) upon running a 50 ng/mL NP calibrator.

To further evaluate the analytical performance of the developed dual-analyte sensor, the cross-reactivity of the specific antibodies for each analyte toward the other was tested. This was achieved by preparing single analyte calibrator solutions and running them over biochips modified with both capture antibodies. As shown in Figure 7, when a calibrator containing 50 ng/mL of NP was run over a chip coated with the anti-NP antibody, a signal of approximately 0.90 nm was obtained. In contrast, when the 50 ng/mL RBD calibrator was run over the same chip, the signal was about 0.04 nm, which was barely distinguishable from the baseline standard variation of ±0.02 nm calculated by running assay buffer over the chip. Similar results were observed when the NP calibrator was run over a chip modified with the anti-RBD capture antibody, indicating the high specificity of the developed dual-analyte assay.

### 3.2. Analytical Characteristics of RBD-NP Immunosensor

**Figure 8:**
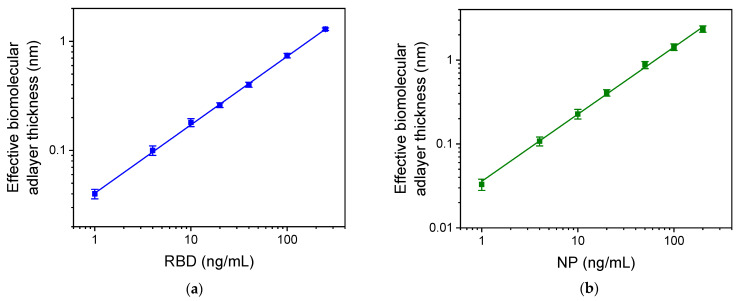
Typical calibration curves for (**a**) RBD and (**b**) NP obtained with the dual-analyte MARS biosensor. Each point is the mean value of three measurements ± SD.

The calibration curves obtained for RBD and NP with the MARS immunosensor are presented in Figure 8a,b, respectively. The linear regression equations of the curves were y = 0.62 (±0.01) x − 1.4 (±0.02), with correlation coefficient R^2^ = 0.9994 for RBD, and y = 0.80 (±0.02) x − 1.4 (±0.02), with R^2^ = 0.998 for NP. The assays limit of detection (LOD) and quantification (LOQ) were calculated as the concentration corresponding to +3 SD and +6 SD, respectively, of 10 replicate measurements of zero calibrator. The LOD was 1 ng/mL and the LOQ was 2 ng/mL for both analytes. In addition, the linear dynamic range extended up to 250 ng/mL for both analytes.

The reproducibility of the dual-analyte assay was determined by analyzing three samples, covering the dynamic ranges of the assays, prepared by spiking different concentrations of RBD and NP in extraction buffer. The intra-assay coefficients of variation (CVs) were determined by performing four repetitive measurements of each sample within the same day. For both RBD and NP, the intra-assay CVs ranged from 3.6% to 8.5%. The inter-assay CVs were calculated based on four measurements of the three samples conducted on four different days, and they varied from 4.5% to 9.8% for both analytes.

To assess the accuracy of the dual-analyte assay, recovery experiments were conducted by spiking known concentrations of RBD and NP in samples prepared in the extraction buffer of the virus from nasopharyngeal swabs, which were provided along with a commercial SARS-CoV-2 self-test kit. The percent recovery was calculated as the percentage ratio of the added concentration to the difference in concentration determined in each sample after and before the addition. The results, presented in Tables S1 and S2, for RBD and NP, respectively, indicate that the mean recovery values ranged from 90.0 to 110%, demonstrating the high accuracy of the developed dual-analyte immunosensor.

**Figure 9:**
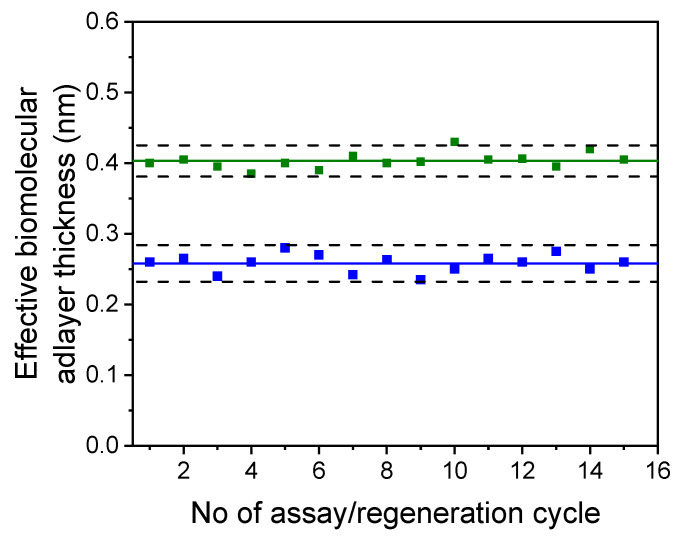
Signal responses obtained from a dual-analyte chip for 15 repetitive assay/regeneration cycles using a calibrator containing 20 ng/mL of RBD (blue squares) and 20 ng/mL of NP (green squares). The solid line corresponds to the mean value of 15 measurements, while the dashed black lines correspond to mean value ± 2 SD for each analyte.

The potential for regeneration and reuse of the biochip was investigated to test the stability of the immobilized antibodies and the repeatability of the dual-analyte immunosensor response. Various solutions, including 0.1 M glycine/HCl buffer pH 2.5, 0.05 M HCl, and 0.05 M NaOH, were tested for biochip regeneration after the completion of the assay. It was determined that the optimal solution for biochip regeneration was 0.05 M HCl. When the HCl solution was run for 3 min after the completion of the assay, no detectable signal was received when the detection antibodies, secondary antibodies, and streptavidin were subsequently run over the chip. By conducting repetitive assay/regeneration cycles, it was found that a single dual-analyte chip could be regenerated at least 15 times without any loss of signal for both analytes (Figure 9).

The long-term stability of dual-analyte chips was also evaluated using biochips prepared in a single batch and kept at 4 °C in the presence of a desiccator for a period of 10 weeks. During this period, assays were run using different biochips, and it was found that the coefficient of variation of the responses obtained was lower than 10%.

### 3.3. Comparison with other Optical Detection Methods

A comparison of the developed MARS biosensor with other optical biosensors reported in the literature for the determination of RBD and NP SARS-CoV-2 proteins is summarized in Table 1. The data presented for each method include the sensing principle, the targeted analyte, the use of labels or lack thereof, the assay dynamic range, and the analysis time. Despite several optical biosensors being reported for the determination of either RBD or NP, this is the first time that an optical label-free biosensor is proposed for the simultaneous determination of RBD and NP, aiming to the detection of SARS-CoV-2. Regarding RBD detection, two of the reported biosensors [34,35] involved labels and, as expected, they provided ultra-high detection sensitivity compared to the label-free ones. Additionally, for one of them [35], the duration of the analysis was not suitable for point-of-care applications since a single measurement lasted more than 1 h. Among the label-free optical biosensors reported in the literature, one was based on the surface plasmon resonance detection principle in the optical fiber format. This sensor provided lower LOQ than that of the developed biosensor for an assay with a duration of approximately 15 min [43]. However, the sensor response was characterized by high standard deviation values that could limit its application in the sample analysis. Another label-free biosensor reported in the literature was based on interference measurements using an imprinted photonic crystal film as a substrate modified with antibodies [36]. The LOQ reported in this work is four orders of magnitude lower than that of the developed biosensor, but the assay duration is approximately 1 h. Moreover, a smartphone-based measurement system has also been investigated for point-of-care analysis, but an integrated device had not been developed, in contrast to the MARS biosensor proposed in this work. Two fiber-optic based biosensors have been also reported for RBD detection. The first one was based on the use of an antibody-conjugated phase-shifted long-period fiber grating (PS-LPFG), claiming a wide dynamic range from 1 pg/mL to 100 μg/mL for an assay duration of 20 min [44]. In this case, as well, the variation of sensor response reported for different calibrators makes the sensor discrimination ability over the claimed concentration range questionable. The second fiber-optic based biosensor was an all-fiber optofluidic biosensor based on the Fresnel reflection detection principle, and it provided a LOQ of 0.01 ng/mL in less than 10 min [45].

Regarding the detection of NP, two label-free optical biosensors have been included in Table 1. In the first one, ultra-fast determination (<5 min) of NP is achieved through a microcantilever sensing platform with a LOQ of 1 ng/mL [37], while in the second one, a microcavity in-line Mach-Zehnder interferometer (μIMZI) was exploited for NP detection with a LOQ of 3 ng/mL for an assay of 30 min [46]. Although the LOQs achieved with the proposed biosensor are not as low as those reported in the literature for other optical biosensors that operated in labeled [34,35] or label-free format [36,37,43,44,45,46], the developed MARS biosensor offers the advantage of simultaneous determination of both RBD and NP in the same sample, which could improve the clinical sensitivity of the method regarding SARS-Co-2 infection detection. Furthermore, the developed biosensor can analyze extracts of nasopharyngeal swabs without further pretreatment within 12 min at the point-of-care, employing the integrated biosensing device.

## 4. Conclusions

In this study, an optical label-free immunosensor based on the Multi Area Reflectance Spectroscopy (MARS) principle has been presented for the simultaneous determination of RBD and NP proteins of SARS-CoV-2. Multiplexed analyte detection is achieved using silicon chips with two sensing areas of silicon dioxide with different thickness, enabling the monitoring of assays performed on each area using a single reflection probe for spectrum recording. The developed immunosensor enables the simultaneous determination of RBD and NP through a non-competitive three-step immunoassay, which is completed in 12 min. This assay allows for the selective and sensitive determination of both analytes, thereby increasing the accuracy of SARS-CoV-2 virus detection tests. The method demonstrates high reproducibility, with intra-assay and inter-assay coefficient of variation (CV) values lower than 8.5% and 9.8%, respectively. Additionally, the mean recovery values ranging from 90% to 110% for both analytes indicated the high accuracy of the dual analyte assay. The immunosensor is combined with an integrated user-friendly device that enables the automated analysis of samples. Calibration curves determined for each analyte have been imported into the software controlling the MARS reader, automatically providing concentration values for both proteins in the samples analyzed after the completion of immunoassays. The method was evaluated using spiked samples prepared in extraction buffer from a commercially available self-test kit for SARS-CoV-2 collection from nasopharyngeal swabs. These samples could be analyzed without any treatment using the calibration curve prepared in the assay buffer. The combination of fast analysis and the good analytical characteristics of the assay, along with the automated operation of the device, make the proposed immunosensor suitable for SARS-CoV-2 detection at the point-of-care. In addition, despite the fact that the COVID-19 pandemic is currently declining worldwide, the sensing platform developed should be a useful tool in the armory against future health threats.

## Figures and Tables

**Table 1:** Comparison of the developed MARS biosensor with the literature’s optical biosensors for the detection of RBD and NP SARS-CoV-2 proteins.

Sensing Principle	Analyte	Recognition Molecule	Label	Dynamic Range	Analysis Time (min)	Ref. No
Fiber-optic biolayer interferometry (FO-BLI)	RBD	antibody	3-amino-9-ethylcarbazole	0.5–16 fg/mL	13	[34]
2D MXene-based SPR	RBD	antibody	PDA-AgNPs nanohybrids	0.1 pg/mL–1 μg/mL	>60	[35]
SPR D-shaped plastic optical fiber (POF)	RBD	aptamer	no	1–40 pg/mL	>10	[43]
Imprinted photonic crystal	RBD	antibody	no	1 pg/mL–100 ng/mL	<60	[36]
Phase-shifted long-period fiber grating (PS-LPFG)	RBD	antibody	no	1 pg/mL–100 μg/mL	>20	[44]
All-fiber optofluidic biosensor (LF-AOB)	RBD	antibody	no	0.01–100 ng/mL	<10	[45]
Microcantilever	NP	antibody	no	1 ng/mL–1 μg/mL	<5	[37]
Μicrocavity in-line Mach–Zehnder interferometer **(**μIMZI)	NP	antibody	no	3–300 ng/mL	30	[46]
MARS	RBD NP	antibody	no	20 ng/mL–1 μg/mL	12	This work

## Data Availability

The data presented in this study are available on request from the corresponding author. The data are not publicly available due to privacy issues.

## Supplementary Materials

The following supporting information can be downloaded at: https://www.mdpi.com/article/10.3390/bios13090865/s1, Figure S1: Image of the compact biosensing device employed for the development of the proposed MARS immunosensor; Table S1: Percent recovery values of RBD added to calibrators prepared in extraction buffer of nasopharyngeal swabs obtained from a commercially available kit.; Table S2: Percent recovery values of NP added to calibrators prepared in extraction buffer of nasopharyngeal swabs obtained from a commercially available kit.
