# Supplementary material for: Simultaneous Detection of SARS-CoV-2 Nucleoprotein and Receptor Binding Domain by a Multi-Area Reflectance Spectroscopy Sensor
Source: Biosensors (Basel). 2023 Sep 1;13(9):865. doi: 10.3390/bios13090865 (PMC10526254; doi:10.3390/bios13090865)
Supplement: Supplementary file 1 [file biosensors-13-00865-s001.zip › biosensors-2554669-supplementary.pdf]

biosensors

MDPI

Supplementary Material

Simultaneous Detection of SARS-CoV-2 Nucleoprotein and Receptor Binding Domain by a Multi-Area Reflectance Spectroscopy Sensor

Dimitra Tsounidi [1], Michailia Angelopoulou [1], Panagiota Petrou [1], Ioannis Raptis [2], Sotirios Kakabakos [1*]

[1] Immunoassays-Immunosensors Lab, Institute of Nuclear and Radiological Sciences and Technology, Energy and Safety, NCSR "Demokritos", 15341 Aghia Paraskevi, Greece; dtsounidi@rrp.demokritos.gr (D.T.); mikangel@ipta.demokritos.gr (M.A.); ypetrou@rrp.demokritos.gr (P.P.)

[2] Institute of Nanoscience and Nanotechnology, NCSR "Demokritos, 15341 Aghia Paraskevi, Greece; i.raptis@inn.demokritos.gr

* Correspondence: skakab@rrp.demokritos.gr; Tel.: +30-2106503861 (S.K.)

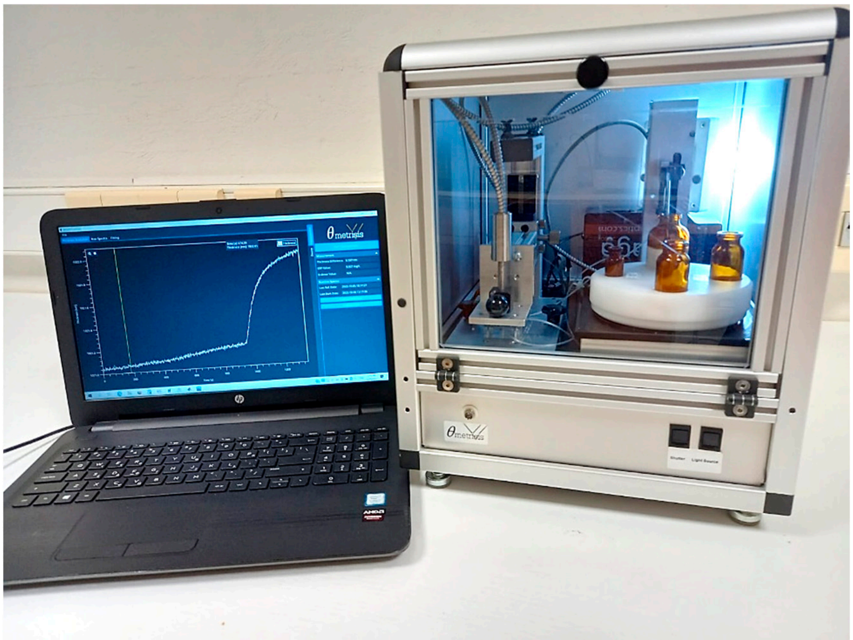

Figure S1. Image of the compact biosensing device employed for the development of the proposed MARS immunosensor.

Table S1. Percent recovery values of RBD added to calibrators prepared in extraction buffer of nasopharyngeal swabs obtained from a commercially available kit.

Sample	Concentration prior to addition (ng/mL)	Concentration added (ng/mL)	Concentration determined after addition (ng/mL)*	Recovery (%R)
1	0	10	10.9±0.5	109±5.0
2	10	10	19.0±0.8	90.0±8.0
3	20	20	41.8±1.4	109±7.0
4	50	50	95.2±2.1	90.4±4.2
5	100	80	185±5.0	106±6.2

* Mean value ± S.D.; N=3.

Table S2. Percent recovery values of NP added to calibrators prepared in extraction buffer of nasopharyngeal swabs obtained from a commercially available kit.

Sample	Concentration prior to addition (ng/mL)	Concentration added (ng/mL)	Concentration determined after addition (ng/mL)*	Recovery (%R)
1	0	10	9.6±0.4	96.0±4.0
2	10	10	20.8±0.6	108±6.0
3	20	20	40.5±1.2	102±6.0
4	50	50	105±2.3	110±4.6
5	100	80	175±4.0	93.7±5.0

* Mean value ± S.D.; N=3.
